# Tracing the Decisions That Shaped the Development of MyChart, an Electronic Patient Portal in Alberta, Canada: Historical Research Study

**DOI:** 10.2196/17505

**Published:** 2020-05-26

**Authors:** Melita Avdagovska, Tania Stafinski, Mark Ballermann, Devidas Menon, Karin Olson, Pauline Paul

**Affiliations:** 1 University of Alberta School of Public Health Edmonton, AB Canada; 2 University of Alberta Faculty of Medicine and Dentistry Edmonton, AB Canada; 3 University of Alberta Faculty of Nursing Edmonton, AB Canada

**Keywords:** patient portals, information technology, decision making, investments

## Abstract

**Background:**

Understanding how health organizations decide on information technology (IT) investments is imperative to ensure successful implementation and adoption. There is a high rate of failure and a tendency to downplay the complexity of implementation progression. Alberta Health Services introduced a patient portal called MyChart. Although MyChart allows patients to view appointments and selected laboratory results and to communicate with their providers, its uptake varies.

**Objective:**

The study aimed to examine the institutional decision-making processes that shaped the development and implementation of MyChart.

**Methods:**

A historical study was conducted based on the 7-step framework, where one engages in a rigorous archival critical analysis (including internal and external criticism) of documents and analysis of interviews. We reviewed and analyzed 423 primary and secondary sources and interviewed 10 key decision makers.

**Results:**

Supportive leadership, project management, focused scope, appropriate technology and vendor selection, and quick decision making were some of the facilitators that allowed for the growth of proof of concept. The planning and implementation stages did not depend much on the technology itself but on the various actors who influenced the implementation by exerting power. The main barriers were lack of awareness about the technology, proper training, buy-in from diverse system leaders, and centralized government decision making.

**Conclusions:**

Organizational priorities and decision-making tactics influence IT investments, implementation, adoption, and outcomes. Future research could focus on improving the applicability of needs assessments and funding decisions to health care scenarios.

## Introduction

### Backgound

Aging populations, increased prevalence of chronic conditions, and consequent rising costs significantly challenge health care systems worldwide. One proposed solution to these challenges has been health information technologies (ITs) that empower patients to be partners in their care, support evidence-based, individualized care, and monitor population health [1]. Understanding how health organizations decide on IT investments is imperative to ensure successful implementation and adoption. There is a high rate of failure and a tendency to downplay the complexity of implementation progression.

The literature shows that the majority of health IT investments are struggling to achieve the hoped-for improvements in quality of care and economic benefits [2-5]. Furthermore, these technologies tend to run over budget because of inadequate preparation for the complexities of implementation [6,7]. There is a need to understand the implementation and adoption of such technologies through a life cycle approach for the technology rather than as a decision at a single point in time. Understanding the events and actors involved in each stage of the cycle provides for better future planning for the successful implementation of IT investments such as patient portals [8]. Alberta Health Services (AHS) introduced a patient portal called MyChart through a proof of concept (PoC).

### Aim and Objectives

This study aimed to investigate and describe the process by which health IT, in this case, a patient portal, was introduced into the provincial health system of Alberta, Canada. The focus was on the process of decision making and the chronological timelines that led to the pilot of the patient portal, with an emphasis on the conceptualization, development, and implementation processes: need (why), process (how), decision makers (who), decision (what), setting and context (where), and timelines (when).

## Methods

### Study Design

**Figure 1:**
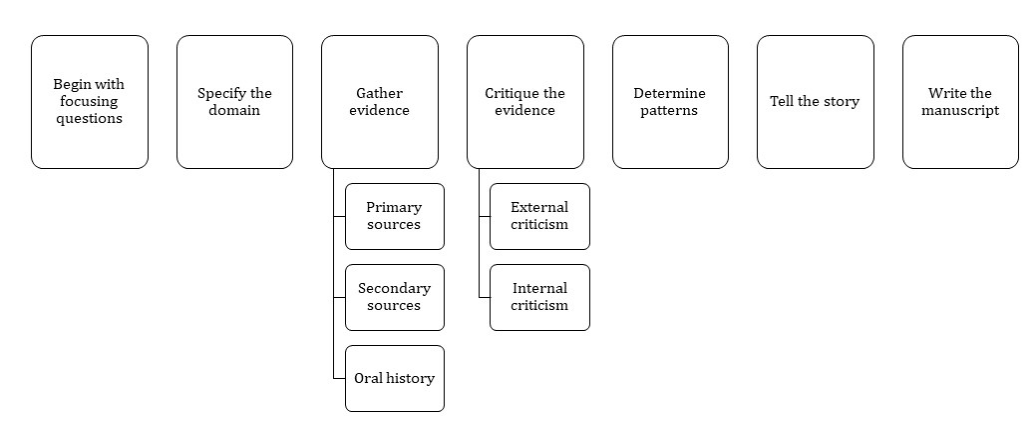
Mason et al (1997) 7-step framework.

A historical research approach was used to trace the history of the development and implementation of a patient portal in several clinics in Alberta [9-13]. Specifically, the 7-step methodology framework developed by Mason et al [9] for studying medical information systems was used, as shown in Figure 1. This entails a rigorous archival critical analysis (including internal and external criticism) of numerous documents (contracts, meeting agendas and minutes, training and marketing materials, reports, decision requests, etc), analysis of key informant interviews, and development of the narrative [9].

Historical research includes the methodical collection and appraisal of data to recognize, understand, explain, illuminate, and accurately reconstruct past events, actions, and decisions [10-12]. Organizations may associate IT solutions with *awesome potential* and lose track of concerns and problems, resulting in repetitive regurgitation of ideas and being victims of IT fads and fashions. Mason et al [9] pioneered a framework to describe how a solution and its identified need may be adopted when it encounters the organizational context, including the connections and roles of change agents working to mitigate resistance. History allows for understanding and acknowledging what has worked and what has not worked previously [9]. It is important to outline that studying IT through the historical method is not about understanding the technology. It is about the connections and roles that impact how the technology is implemented and eventually used [9]. The framework developed by Mason et al [9] allows for an in-depth understanding of the organization’ s current practices, how cultural and environmental conditions impacted the decisions, how the need and the problems were identified, shedding light on the resistance, the process of change, and the actors that led the change.

### Study Setting

Alberta Health (AH) is the Alberta government department accountable for ensuring the delivery of health care services and setting and assessing compliance with policy and legislation [14]. Health care service delivery is funded through AHS and primary care networks (PCNs). PCNs coordinate the delivery of primary health services [15,16]. AHS was established in 2008 and delivers care through 400 facilities throughout the province [17]. The focus of this historical study is the MyChart PoC that took place in Edmonton, Alberta.

### Study Context

Planning for a patient portal in Alberta began in the early 2000s led by AH. However, it was not until 2016 that one was introduced into the province, with the launch of a PoC study of MyChart (AHS branding of EpicCare Ambulatory from Epic Systems, 2014 version) by AHS. MyChart was developed by Epic and customized to meet the needs of AHS, allowing patients to view appointments, medical test results, and medication therapies, and to communicate with their providers. It is connected to the central AHS electronic medical record (EMR), named eClinician.

This study received ethics approval from the Research Ethics Board at the University of Alberta (Study ID: MS1_Pro00072286).

### Search Strategy

Information on the introduction of patient portals in Alberta was gathered by first identifying relevant sources. Primary sources, including oral histories (key informant interviews), are materials that provide firsthand accounts of the event of interest [18-20]. Oral histories are considered a primary source, as the interviews are *for the record* and tend to confirm the events outlined in written documents [21]. Secondary sources are reports, materials, books, or articles written on the topic of interest by people who were not directly involved [10,21]. They provide additional *depth and meaning to a topic* [22].

We developed a search strategy to identify any sources of the development and/or implementation of a patient portal in Alberta. As the implementation of patient portals was conducted internally, government archives were searched in February 2018, and it was identified that AHS and Alberta Health maintained the sources. Requests were submitted, and the AHS project leadership agreed to provide the documents. In addition, MA (lead researcher) contacted the provincial and AHS archive departments, but the archivists found no documents on this topic. It was expected that most of the written data sources (both primary and secondary) would be internal documents; thus, an agreement was signed with AHS for document access.

In addition, we conducted a Web-based search of academic electronic databases (PubMed [MEDLINE and non-MEDLINE] references and Cumulative Index of Nursing and Allied Health Literature ). The search focused on the term *Alberta patient portal* with the intent of identifying articles that had any descriptions about the development of a patient portal in Alberta.

### Sampling Procedure

Purposive sampling was used to recruit key informants from the AHS and AH. The names of possible participants were identified through meetings with AHS and Alberta Health representatives and a search of government directories. Both Alberta Health and AHS individuals were interviewed because of their role in planning for a patient portal. This sampling approach is common in historical studies, as the researcher requires information from individuals with firsthand knowledge of the topic under investigation. Furthermore, this approach was *selected because of the representativeness and uniqueness of their experiences, not because of the generalizability of the findings* [23].

Potential participants were sent an information letter and consent form, giving full details of the study. Once a contacted individual had agreed to participate, an interview was scheduled with each person individually. In total, 19 individuals were approached, and 10 agreed to participate in a semistructured interview. All data were analyzed and reported anonymously. In order to ensure that the participants’ current positions were not jeopardized because of the opinions they offered, the names and specific positions of participants were kept confidential.

### Data Collection Procedure

#### Selecting Relevant Sources

We considered documentary sources relevant if they described any information about patient portals in Alberta. They included project management documents, scope or function documents, decision requests, presentations, organizational charts, user manuals, privacy documentation, meeting minutes and follow-ups, contracts, briefing notes, and correspondence. We also included sources that discussed electronic health records (EHRs) or EMRs systems and potential vendors. Sources that were not related to patient portals and/or EHRs or EMRs were excluded. A total of 423 sources were included.

Documents underwent external and internal criticism by MA and were reviewed by PP (co-researcher). External criticism considers the validity of the documents by confirming where the document came from and who had documented the fact that the source existed. Internal criticism looks within the data itself to try to determine truth, even considering the motives of the person providing the data [21]. This process of trustworthiness and credibility was performed for each retrieved source [22,23].

#### Interviews

The interviews were conducted face-to-face or by telephone by MA and were recorded and transcribed *verbatim*. Participants were given an alphanumeric label to protect their identity. The interviews provided an opportunity to clarify various written sources and eyewitness accounts. A generic description of the positions of the key informants is presented in Table 1.

**Table 1:** Organizational designation of key interview participants.

Organization and designation of key interview participants	
Alberta Health	Executive-level participant 1 Executive-level participant 2 Executive-level participant 3 Senior-level participant 4
Alberta Health Services	IT senior-level participant 5 Senior-level participant 6 Executive-level participant 7 Senior-level participant 8 Senior-level participant 9 Clinician senior-level participant 10

Each interview lasted for 45 min and 1 hour. The interview sessions began with clarification of the objective of the study, and a description of the information was sought. The interview questions and discussions focused on the need for a patient portal in Alberta, intended outcomes, facilitators, and barriers to the design and implementation processes, stakeholders, policies and legal factors, and future recommendations.

### Data Extraction and Synthesis

The sources were numbered from 1 to 423 for ease of data extraction and referencing. We developed a data extraction form to record any information about the development and implementation processes of any patient portal in Alberta. The final form included items related to source type, date of creation, author position and affiliation, summary of source information, impact of source, possible quotes, and related sources. The primary and secondary sources were grouped by year (2005-2019) and type (planning, decision requests, agendas, minutes, presentations, contracts, scoping documents, and optimization documents). MA conducted the data extraction overseen by PP.

The transcription of the interviews was done by MA and overseen by PP and KO (co-researcher). In historical research, interviews are not analyzed to develop themes but to juxtapose the insights provided by the oral interviews with the *official* documents. The interviews were entered into NVivo (version 11), which was used to track the analytical process through memos and notes. Interview data extraction related to events and actions was connected with patient portals. In addition, potential quotes were identified as being related to various events.

## Results

### Data Collection Overview

**Figure 2:**
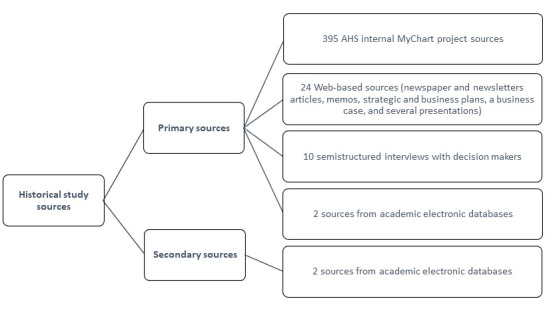
Overview of data collection strategy.

Figure 2 shows the number of selected primary (including interviews) and secondary sources.

The following sections describe the events based on the extracted data from these sources.

**Figure 3:**
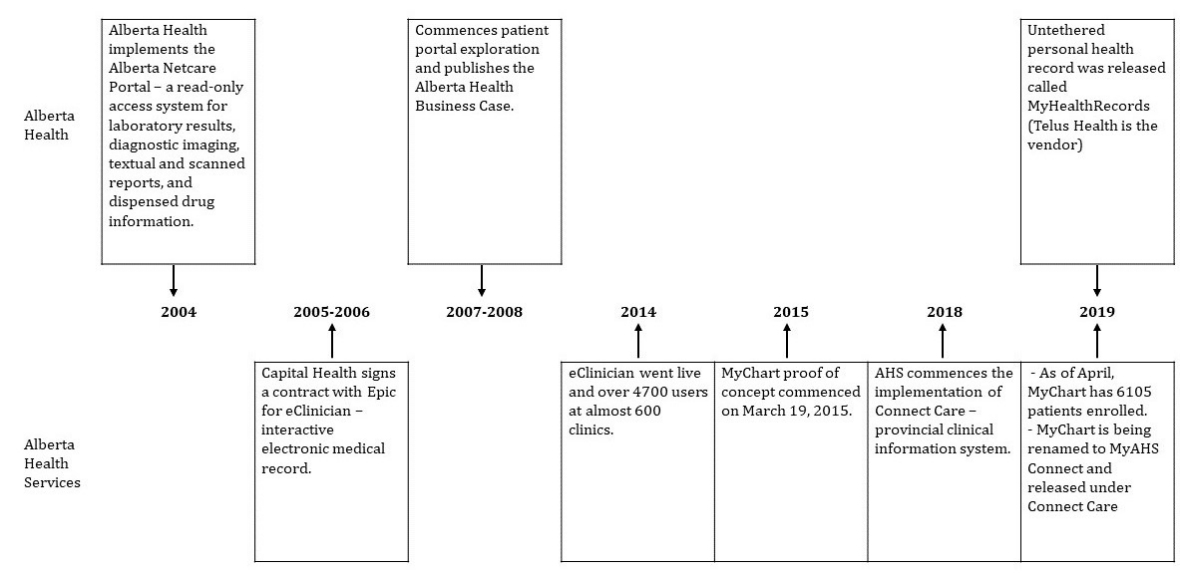
Timeline of the development of patient portals in Alberta. EMR: electronic medical record.

An overview of the Alberta Health and AHS timelines is shown in Figure 3.

### The Unmet Need

#### Before 2005

Alberta’s first EMR implementation planning began in 1997 (before the creation of a single province-wide health services delivery organization) with the directive to develop and deliver a single province-wide EMR [24]. This led to the implementation of the Alberta Netcare Portal (ANP) in 2004, which was a read-only access system for laboratory results, diagnostic imaging, textual and scanned reports, and dispensed drug information [25].

#### 2005/06: Capital Health and the Need for a Different Electronic Medical Records

In 2005, Capital Health (1 of the 7 regional health authorities at that time) issued a request for proposals (RFPs) for a different EMR system to replace the ANP system. Epic Systems Corporation (from now on referred to as Epic) was awarded the contract in September 2006. The new EMR system, which was developed and customized for Alberta, was called eClinician.

#### 2007/08: The Alberta Health Business Case

Although Capital Health and later AHS were working on implementing eClinician, AH commenced a patient portal discovery phase based on reports from the United States about the potential for health ITs to be cost saving (business case: personal health portal [Advice to the Minister]). This led Alberta Health to develop a business case that represented the first official document produced by a government body in Alberta that described the need for patients to have access to their personal health records. In addition, patients were *falling through the cracks*, as they were unaware of their test results or if specialist referrals were being made (AH executive-level participant 4).

Although in 2008, AH planned to have a portal deployed within a few years, this did not occur until 2019. Several executive leaders confirmed a description of events during this time when delays resulted from inappropriate planning, changes in vendors, and changes in AH leadership. In addition, individuals involved in managing the personal health record (PHR) project were not equipped with the appropriate technical knowledge as described by an interview participant:

So what happened was we ended up in the development mode we’re not in the software business. We are not in development business.

AH executive-level participant 1

The portal planning undertaken by AHS during 2008 was also seen as a reason for delays:

I also think that the culture of AHS has worked against us because they didn't want something separate from their clinical information system. They wanted it to be something that they controlled and part of the software that they would be purchasing. I believe that that culture and that resistance was evident through the whole journey.

AH executive-level participant 1

It appears from the key informant interviews and documents that between 2005 and 2008, numerous activities established foundational components for a patient portal in Alberta. It is difficult to judge whether the commitment to meet the identified technology need was rushed or whether the task was more complicated than expected. It took more than 11 years for AH to finally release a PHR system in March 2019 [26]. During these years, an apparent or actual lack of coordination between AH and AHS resulted in tension due to their two patient portal systems appearing duplicative, being established within the same health care system.

#### 2014-2019 Alberta Health Services and the Race to Deliver a Patient Portal

The delays that occurred between 2008 and 2014 were caused by the restructuring that occurred in Alberta when 12 separate health regions and 3 health boards were merged into Canada’s first province-wide, fully integrated health system known as AHS. The AHS’ patient portal journey was a continuation of what had been initiated by Capital Health (one of the former health regions) by its implementation of eClinician. As AHS was planning the implementation of a provincial inpatient clinical information system, eClinician was meant to serve as a bridge that would ease the gap in terms of the identified need for a provincial EMR and an interactive way for patients and health care providers to access information. In 2014, the eClinician Working Group, with input from various stakeholders, developed a document outlining the AHS health information–sharing prioritization principles that described the need for cost-saving measures based on patients having access to their health care information. In 2014, eClinician went live with over 4700 users at almost 600 clinics or sites [27]. It is important to point out that the AH ANP EMR was still being used in Alberta during the implementation of eClinician.

The eClinician system was intended to support *one person, one record,* and had the capacity to deliver the MyChart patient portal through which patients could access their record, communicate with their provider, and book and cancel appointments. The MyChart PoC, led by a clinical Working Group (MyChart Working Group) reporting to the AHS Ambulatory Oversight Council, was planned in phases that were meant to reflect a forward-thinking plan to align with both the AH PHR and the upcoming AHS Clinical Information System.

As a small project team what we did is we created a Clinical Guidance Working Group it was called the MyChart Working Group and on there we had a number of different users on board. We had physicians, nurses, allied health, from different sites and we had managers on there as well.

AHS senior-level participant 8

The MyChart PoC implementation was planned as an incremental change in order to minimize resistance by clinicians and patients (eClinician prioritization principles). It was decided that at the conclusion of the PoC, the Working Group would validate the solution against physician's expectations, assess the true performance of the patient portal in a controlled environment, identify areas for improvement, identify implementation tips and traps, and lessons learned, measure key performance indicators and determine the return on investment (eClinician link⸺PoC scope August 21, 2015).

The results from the PoC would be used to determine any future approaches for deployment, long-term expansion, or modifications of the health IT. Thus, between 2014 and 2019, AHS planned, developed, customized, and implemented a patient portal—a process that has been characterized by intricate agenda-setting and decision-making processes. The following sections will describe the technology and what needed to be done to commence the PoC and the subsequent successful implementation and adoption.

### MyChart: The Change

The AHS MyChart was a customizable Web app that offered patients easy access to their medical records via controlled access to the same eClinician medical records used by their physicians. It provides self-service functions that have the capacity to reduce administrative costs and increase patient satisfaction. It also offers various features that organizations can select to meet their identified requirements (MyChart Recommendations: Core Features). Each function is implemented based on the need and cost.

Patients participating in the MyChart PoC were able to view their health summaries (problem list, medications, allergies, and immunizations), laboratory and diagnostic imaging test results, previous and upcoming appointments, and letters sent from clinics they have attended. In addition, patients were able to send nonurgent messages to their health care team, request appointment dates and times, complete health assessment questionnaires, and enter information (ie, vital signs or blood glucose measurements).

**Figure 4:**
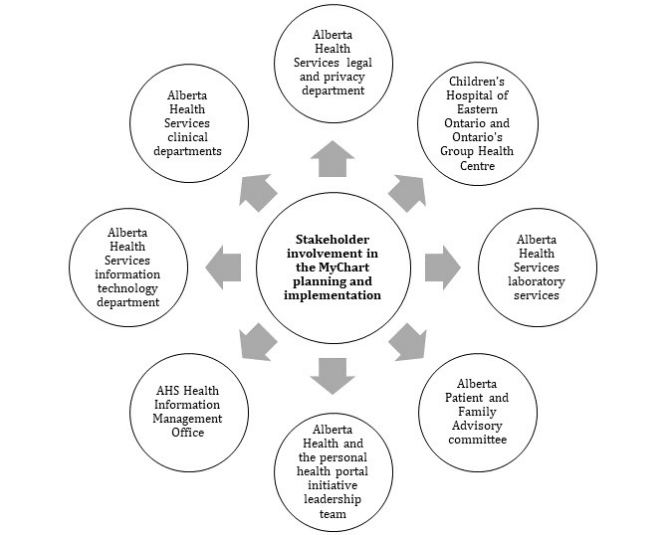
Stakeholder involvement in the MyChart planning and implementation. AHS: Alberta Health Services; IT: information technology.

Before deciding on how the PoC will evolve and if MyChart was the right technology, the work was precipitated by extensive engagement with various stakeholders, as described in Figure 4 (MyChart summary report). This wide consultation process allowed the MyChart Working Group to build credibility in the work they undertook.

**Figure 5:**
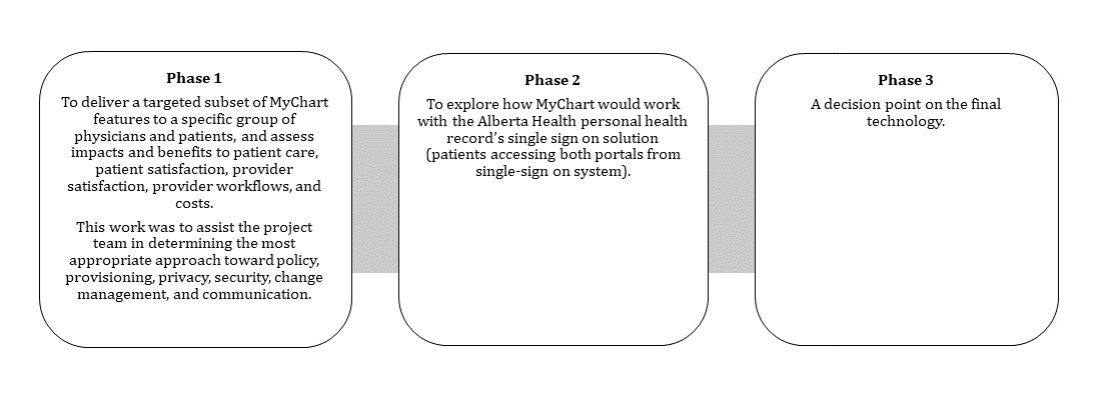
Planned MyChart proof of concept phases. PHR: personal health record.

Before the PoC started, the MyChart Working Group had to plan and consider all possible variables, tasks, and situations that might arise, and this work was guided by the Epic team (weekly status report for eClinician foundation—eClinician MyChart). The planned PoC phases are shown in Figure 5.

The PoC was deployed by ensuring that the best *bang for our buck* was achieved by only working with health care providers that were willing to be part of the study and implementing functions that were going to be utilized (MyChart summary report). The vision for MyChart PoC was *better health, powered by information, supported by technology* (MyChart monthly CIS project status report, August 2015). The estimated PoC implementation cost (more than US $873,600 ) was based on the cost of staff to support the PoC, and the purchase of the required software and hardware (MyChart PoC scope, September 9, 2015). By April 22, 2015, the PoC was up and running. The initial goal was to engage 500 patients participating across clinics, with significant measurement and benefits realization activities.

#### Making the Case for MyChart

The launch of the MyChart PoC required several components that were determined to be crucial for success, such as the recruitment of clinics and health care providers, customization of the portal, and an understanding of how MyChart would align with the AH PHR initiative.

The MyChart Working Group established project management principles to guide them through the planning and implementation process, guided by charter principles as shown in Textbox 1 (eClinician: Lessons learned in context MyChart project).

**MyChart proof of concept charter principles.**

- Keep it simple
- Keep the focus on the patient
- Have clear communication with each other and with the task force group
- Let us work together, collaborate, and take joint responsibility for getting it done (no finger pointing)
- Listen to Epic! (Let us not waste time reinventing the wheel, Alberta Health Services is not all that different)

In making the case for MyChart, the MyChart Working Group outlined several cost-saving assumptions, such as a decrease in no-show appointments due to MyChart reminders and the opportunity to implement Web-based scheduling. In addition, with secure messaging, there would be less need for mailing letters to patients, and fewer visits to the emergency department for nonemergent issues, as patients would have immediate access to their health care team. Given that patients would have access to their medical records and test results, the number of follow-up visits was also expected to decrease (eClinician MyChart PoC).

Costs were calculated for the license fees and maintenance of the MyChart app. For each new user who accesses MyChart at least once, there would be an ongoing cost of US $4.05 per year (eClinician MyChart PoC). These costs were offset by the savings realized through the avoidance of unnecessary medical visits.

The Working Group members considered the MyChart PoC as a *bottom up initiative* based on the notion that clinicians had asked for their patients to have access to their medical records (MyChart Epic care AMH, December 3, 2015). Furthermore, the process of *customizing* the patient portal from the American version to a version that worked within the legislation and privacy requirements in Alberta was a long process. There were many decision requests to change how the processes and functions worked, with the intent to create a system that was patient centered and would meet all legislative requirements of Alberta’s Health Information Act.

#### Gaining Support from the Providers

As described by AHS senior-level participant 9,

because we wanted to be successful, we sort of chose a group of willing clinics and physicians who we knew were knowledgeable, that reassured us that the clinic was committed and that they were willing to help us. The Working Group didn't want to force anybody that did not want it to participate in the PoC.

AHS senior-level participant 9

The recruitment of participating health care providers was a challenging process due to the uncertainty of what a patient portal would mean for them:

There were some physicians who were absolutely horrified at the idea of patients being able to view their own results and their own information especially in the case where some decisions were made to release lab results immediately. They were horrified at the idea that the patient might see the results before the physician had a chance to see the result.

AHS senior-level participant 9

Health care providers were concerned because MyChart would not replace anything but instead add to the workflow. The MyChart Working Group wanted to ensure that the initiative would be seen as useful and necessary for the enhancement of patient-centered care. The Working Group decided that the best way to commence the PoC was to find health care providers who were given the autonomy to decide which patients were appropriate candidates for MyChart.

During the promotional and exploratory processes for participating clinics, the Working Group selected 5 clinics in Edmonton to participate in the PoC, as shown in Textbox 2, and offered the opportunity to the health care providers from these clinics to participate or not to participate in the PoC. As of April 2019, MyChart was used at 10 clinics.

**MyChart proof of concept participating clinics.**

- Lynnwood Family Medicine Clinic
- Alberta Health Services (AHS) Inflammatory Bowel Disease (IBD) Clinic
- AHS Kaye Edmonton Clinic (KEC)⸺Multiple Sclerosis
- AHS KEC⸺Diabetes
- AHS KEC⸺Rheumatology

#### Navigating Between the Two Patient Portals

The leading concern about the future of MyChart and the need for a PoC hinged on processes related to deciding how the AH and the AHS patient portals were ultimately going to *work together*:

The AH PHR is essentially access to read-only database of health information. If you want to break it down to most basic stuff, PHR is for reviewing health information, while MyChart is a tool for viewing health information. These are all things that the PHR can never do. It is helpful to say that they are two different things, the problem is that if the patients are coming into MyChart and they can do all these things, why would they bother going to the PHR? That's the problem.

AHS IT senior-level participant 5

As the introduction of the MyChart PoC idea, tensions between AH and AHS in terms of the portal delivery have increased, leading to a *competition-type* delivery of service, as the two systems appeared duplicative.

There were a lot of pull and retain, and a lot of political sensitivities given the amount of money that Alberta Health, the time and effort and money had invested in the PHR. So to get the MyChart® pilot going, we had to escalate it up out of our zone to the IT leadership and medical leadership at AHS first. Then, we had to get permission from the deputy minister level of Alberta Health to do our pilot. We weren't allowed to advertise.

AHS executive-level participant 7

From the early planning days of the MyChart PoC, it was determined that the MyChart portal provided more functionalities than the planned PHR.

We knew that we could offer more complete functionality using the Epic MyChart, basically out of the box. So the public portal that they're building, there was a lot of configuration, they had to build the software. There was a lot of configuration to that kind of thing. And then the software platform that they were building on it was no longer being supported by Microsoft. So we knew that we could surpass their functionality out of the gates in some ways using eClinician MyChart.

AHS executive-level participant 7

Regardless of what was going to be decided, there was a feeling that AH has had the opportunity since 2009 to deliver a patient portal. The sentiment expressed by some participants was that money has been spent, and patients were yet to have access to their health care records. MyChart was seen as a portal that had the potential to be delivered on the identified need.

And for clinicians and patients alike this is a real game changer. There's something that really changes the way we operate in health care. It takes responsibility for health care and it gives it to me, to the patients, they can take responsibility for their own health care, which in theory is everything driven by the physician and dictated by the physician in terms of why he thinks the patients need to know and understand.

AHS senior-level participant 9

The planning for MyChart had to incorporate components as to how the two portals will eventually work together. 

#### Proof of Concept Scope and Functions

The most challenging component of the implementation process was determining the MyChart functions, as there were many options but limited funding and personnel. Organizational policies had to be established for a help desk, customer service requests, key performance indicator report analyses, a MyChart utilization dashboard, and a *go-live* strategy (Epic MyChart project plan, February 2, 2015). There were many functionalities from which to select; therefore, the Working Group had to decide what was important during the PoC.

Then, 1 participant described:

we just sat down in committee meetings and went through the potential futures and whether it was feasible and useful and did we want. And that's how we kind of made our decisions.

AHS executive-level participant 7

The Working Group focused on developing the sign-up process and implementing the following functions: two-way communication, real-time scheduling, release of test results, proxy access, notifications, appointment scheduling, and questionnaires, as described in Table 2.

Decisions on how to proceed were made collaboratively as the Working Group saw this as an opportunity to bring a patient portal in Alberta. As 1 participant described:

so it is this step wise process that we went through and we just sat down in committee meetings and crack through the potential futures and whether it was feasible and useful and did we wanted and our environment. And that's how we kind made our decisions

AHS executive-level participant 7

Therefore, some functions were selected, whereas others were not. Furthermore, when the MyChart PoC was planned, it was decided that the goal would be to offer MyChart to about 500 patients. However, as the PoC evolved and more health care providers decided to participate, it became clear that the milestone of 500 patients was no longer adequate (eClinician MyChart PoC). After considering the positive feedback to date, the AHS agreed to allow for the number of patients participating in the MyChart PoC to be increased up to 5000.

Although there were a great number of anticipated challenges with bringing a patient portal in Alberta, AHS and the MyChart Working Group concluded that not proceeding with this PoC was not an option.

**Table 2:** Planning and development of functions during the MyChart proof of concept.

Function	Planning and development process
Sign up process	The sign-up process was a source of significant grievances by patients and health care providers, “because we put so much security on it and made them use passwords, we made it quite complex” (AHS clinician senior-level participant 10). A two-factor authentication process was designed to comply with privacy legislation. Some never tried to create a MyChart account again after an unsuccessful initial attempt. When MyChart was first introduced, the sign up was based on a process whereby a provider would print a letter containing a MyChart activation code, and then the patient would take it home and sign up for the account at his or her convenience. Although this process seemed simple, many patients had issues with the code, remembering to set up the account, or losing the printed paper. There were numerous discussions on how to improve this process and make it more efficient (MyChart activation workflows—pros and cons). After considering the various options for sign up and soliciting advice from Epic, the MyChart Working Group decided on an email process. Once a provider had introduced and discussed MyChart with the patient, and he or she agreed to sign up, the provider clicked the MyChart status icon on the patient header in eClinician [Decision Request 11: Create option for MyChart activation letter to be sent to patients via email]. This initiated an email containing an activation letter that was directly sent to the patient’s email address documented in MyChart. The email included general instructions about MyChart and the patient’s MyChart activation code. The patient had to enter his or her personal health care number, date of birth, and activation code. The sign-up process included attaching a label to all patients offered MyChart, which indicated whether they were *active* (account used on regular or as needed basis), *inactive* (patient signed-up but never used), *pending* (a patient starts the sign-up process but does not complete it), and *declined* (patient declines to sign up). These labels were as a way of keeping track of patients (Decision Request 238: Add MyChart status to patient header).
Two-way communication	MyChart included two-way patient-provider secure communications for nonemergent issues. This function required many modifications and decisions because it had not been tested before the PoC, and participating clinicians had expressed skepticism. It was also one of the main reasons many health care providers within the participating clinics decided not to participate in the PoC. Providers assumed that they would be inundated with a large number of messages, and there was no compensation plan in place to remunerate them for their time. This function was seen as time saving not only for providers but also for patients. To effectively evaluate the two-way communication, clinics had to decide on the message routing, and how responses would be managed, although the clinics agreed to a process, and each adapted to meet their needs. In some clinics, clinicians monitored the messages directly, whereas in other clinics, nurses or the front office staff managed messages.
Real-time appointment scheduling	For the real-time scheduling function to be applied, participating clinicians had to enter their availability in the system, which would allow patients to select from the available slots. Once a patient selected a slot, the clinic’s office would receive a message. If the booking was done incorrectly or the slot changed, the office staff would call the patient and modify the booking. This function allowed patients to not only book appointments, but also cancel them at their convenience. Although this function was available to all clinics during the PoC, only 1 clinic (community-based family clinic) gave their patients access to real-time scheduling.
Release of test results	Release of test results required the lengthiest deliberations because it needed to comply with the legislative requirements of the Health Information Act. Three options were discussed: auto release (immediate release of all nonsensitive results), time-delayed auto release (some results to be released after a 7-day delay), and no release (results of sensitive tests with the potential for security and privacy concerns). Each was explored, although Epic recommended automatically releasing as many results as possible, the MyChart Working Group decided that results would be released after 10 days. Patients would receive an email notification once the results were posted in MyChart.
Proxy access	Alberta Health Services determined that although proxy access was challenging due to the Health Information Act, this function would be piloted. Proxy access allowed MyChart users to permit others to access their MyChart record by establishing a proxy relationship (eg, parent to child, adult to elderly parent). Proxy access was seen as *breaking new ground* and therefore required careful monitoring and clear guidelines. “So proxy was essentially a precedent issue,” as no service provided by Alberta Health Services had this available electronically (AHS IT senior-level participant 5). When initially introduced during the PoC, proxy access was granted through a manual paper-based process that was found to be very cumbersome and time-consuming. It was later replaced by an electronic proxy access via eClinician. Another issue was that in order to receive proxy access to another patient’s MyChart account, the designated proxy had to first have their own personal MyChart account. This was not always possible, as many family members requesting proxy access were not part of the same clinic that was on eClinician. The Working Group established a *shell* MyChart account containing no personal health information for those that have been approved proxy access to another patient’s MyChart account, but do not have their own MyChart account (AHS IT senior-level participant 5). Although clinics found the proxy sign-up process cumbersome, the benefits of having proxy access appeared to outweigh these concerns, as patients reported positive benefits.
Questionnaires	Although several participating clinics wanted to utilize MyChart for questionnaires and surveys, the MyChart Working Group decided for only 1 specialty clinic to proceed with this function. It was agreed that if this function was deemed successful, it would be recommended for wider implementation.

## Discussion

### Principal Findings

We have presented an overview of the various environmental and contextual factors that influenced the MyChart PoC during the planning and implementation stages. The MyChart PoC was a complex undertaking of design transformation, adjustment, implementation and deployment, collaboration and compromise, and problem solving with the intent to learn about the necessary elements needed to ensure successful wider implementation. The PoC leadership team agreed that the “technology was the easiest part,” as they focused on foundational features with very minimal customization (AHS IT senior level participant 5). The problem-solving processes were impacted by various environmental, social, and professional factors.

Despite extensive research in the area of patient portals, implementation and subsequent adoption of these systems is a convoluted process, which tends to impact settings and practices in different ways [28]. This complexity is clearly shown in MyChart PoC. Furthermore, regardless of the type of setting, these systems require a substantial investment of time, resources, and determination, and not all care settings have the same capacity to contribute equally. As the PoC proceeded, the Working Group saw a difference in the enrollment numbers between the clinics that alluded to differences in uptake.

**Figure 6:**
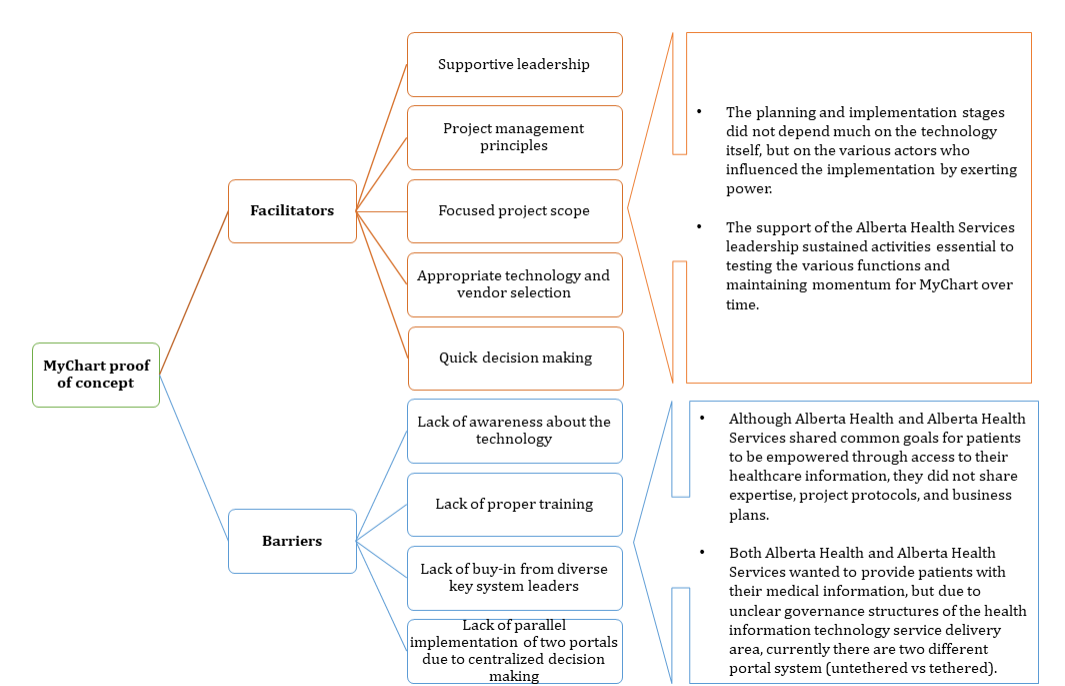
Facilitators and barriers that impacted the MyChart proof of concept during the planning and implementation stages. AH: Alberta Health; AHS: Alberta Health Services.

Through this research study, we wanted to determine what it took to implement a patient portal and what were some of the elements that are necessary not only for successful implementation but also for the successful adoption and optimization of the technology. The review of primary and secondary sources and insights from the interviews explicated several facilitators and barriers that affected the process, as described in Figure 6.

### MyChart Proof of Concept Facilitators

Supportive leadership and quick decision making, project management and focused project scope, and appropriate technology and vendor selection were some of the facilitators that allowed for the commencement and growth of the PoC.

#### Supportive Leadership and Quick Decision Making

The MyChart Working Group comprised leaders who had the capacity to make decisions in support of making the implementation more manageable and without unnecessary delays. It was not only that the leadership was effective, but they also used real and relevant cases to make the case for the portal and garner support. These observations were also confirmed by an interview participant:

I found that in some way, so from concept to deployment it took us what around 6 to 8 months with MyChart. And the reasons I think we were successful is we had strong leadership at the level of the MyChart pilot. And there was medical leadership but also operational leadership. We explicitly had use cases. So we developed a little framework about what are you actually going to use it for, explicitly. That is very helpful and having understanding of the high level concepts. And then we were prepared to make quick and rapid decisions and live with those decisions. And we had clinical input to help us make the decisions.

AHS executive-level participant 7

Leadership was able to try improvements and become leaders in innovations, as they had

own internal executive approval to kind of go for it and try new things and create some precedent.

AHS IT senior-level participant 5

Furthermore, the working Group

had to set the stage when it came for the precedent setting stuff since they considered this project as a PoC and they were given a little bit more leeway.

AHS IT senior-level participant 5

The project leadership was able to create a pull for the technology and created an environment where the demand was greater than the supply.

AHS IT senior-level participant 5

The findings show that the planning and implementation stages did not depend much on the technology itself, but on the various actors who influenced the implementation by exerting power.

The proof of concept was ultimately a learning exercise.

AHS IT senior-level participant 5

As described by AHS executive level participant 7:

We were successful because we had strong leadership at the level of the MyChart pilot.

The support of the AHS leadership sustained activities essential to testing the various functions and maintaining momentum for MyChart over time (eClinician: Lessons learned in context MyChart project).

#### Project Management and Focused Project Scope

The MyChart Working Group developed the PoC based on project management principles. These principles were upheld through various meetings and discussions. Furthermore, the Working Group developed many discussion documents to identify the most suitable scope for the MyChart PoC. As confirmed by AHS IT senior-level participant 5:

first got scope, and once we got clear understanding of who is going to be involved, resources. So scope, resources, finance, and then we could build the schedule. That schedule looked at basically at half dozen clinics and we rolled out everything in a very short period of time.

#### Appropriate Technology and Vendor Selection

The MyChart Working Group saw Epic as the necessary guide in this PoC as they possessed the skills and knowledge on how to implement this type of technology. The assumption was that Epic had done this in many different settings and they had the expertise to understand what was required and what AHS needed to do in order to have a successful PoC. The relationship with Epic was not only for the implementation of eClinician, but also for future partnerships.

So it wasn't really because of the former relationship with them on eCLINICIAN deployment on anything like that, it was more of a perceived ability for them to help us in the future as a partner to deploy the full Connect Care suite of options.

AHS executive-level participant 7

The partnership between the MyChart Working Group and Epic has continued and grown.

### MyChart Proof of Concept Barriers

The main barriers were lack of awareness about the technology, proper training, buy-in from diverse key system leaders, and parallel implementation of two portals due to centralized government decision making.

#### Lack of Awareness About Technology

Although the project team decided not to impose the MyChart PoC on any clinic, various documents show that there was a substantial “lack of awareness*”* (AHS senior-level participant 6) about what patient portals are and what they would do.

There were barriers relating to communicating with the clinics and getting buy in. There was the fear that getting patients their own information would lead to increased work for the physicians in particular. [AHS executive-level participant 7]

Therefore, there was a fear

that patients wouldn't be ready, would get really scared and this would cause a lot of extra work for physicians.

AHS executive-level participant 7

#### Lack of Proper Training

The MyChart Working Group acknowledged that the clinic staff should have received more detailed training. Clinicians perceived MyChart as an add-on and thus did not see training as something needed. This issue was amplified by the fact that the Working Group, due to the small funding, did not have the capacity to establish dedicated staff to assist with any technical and user issues. Although training was not deemed as valuable, as the PoC progressed, clinicians and other clinic staff realized the need for proper training on how the portal works and how patients interact with the system.

#### Buy-In From Diverse Key System Leaders

Buy-in was a challenge due to

fear that giving patients their own information would lead to increased work for the physicians in particular.

AHS executive-level participant 7

Furthermore, there was a lack of different key system leaders and champions. The MyChart Working Group comprised influencers, but they needed additional leaders to expand their spheres of influence.

I think you need for any successful innovation you need champions both within and outside the system but within the system is absolutely critical and fundamental. If you don't have internal champions, it's not going to go anywhere. And I think you really need in most instances health clinician champions.

AH executive-level participant 2

Leadership change also affected the MyChart PoC in negative ways. There were several documents called *handover succession checklists*, which described the complex process of anytime individuals changed (Enhancement PoC eClinician portals: MyChart and eClinician link—Handover succession checklist).

So when you have senior leader change in charge of a project that makes for a challenge as well in terms of transition thing.

AHS clinician senior-level participant 10

#### Parallel Implementation of the Two Patient Portals

**Figure 7:**
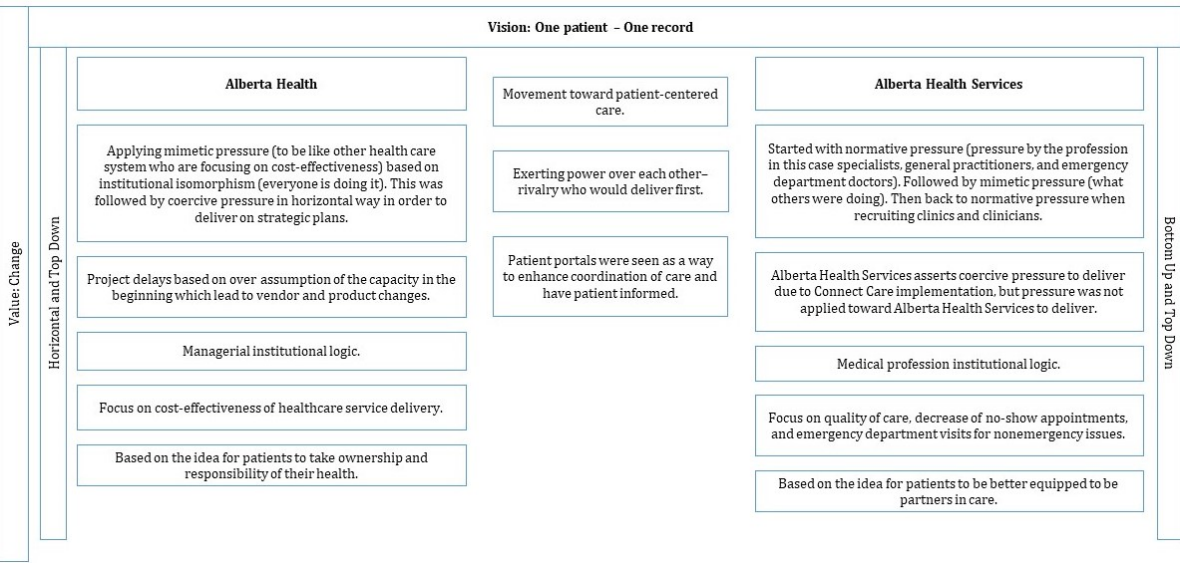
The vision to change. AHS: Alberta Health Services.

Although AH and AHS shared common goals for patients to be empowered through access to their health care information, they did not share expertise, project protocols, and business plans. In addition,, the two approaches described in Figure 7 show the impact of the parallel implementation of the two portals.

As described by an interview participant:

there are two because neither have fully developed themselves out to be the one that is preferential and two, not everyone was involved.

AHS senior-level participant 6

Convergence of the two portals seems to be a natural progression, described as follows:

Convergence has to happen. Someone somewhere eventually has to figure it out.

AHS executive-level participant 7

Both AH and AHS wanted to provide patients with their medical information, but due to unclear governance structures of the health IT service delivery area, currently there are two different portal systems (untethered vs tethered) [29]. These bureaucratic governance complexities will continue to impact how services are planned and delivered in Alberta until there is a better governance structure [30].

The MyChart PoC facilitators and barriers show the complexities that exist within the decisions to implement health IT within different health care settings.

### Comparison With Current Literature

Several studies have focused on identifying facilitators and barriers to patient portal implementation, but only a few have touched on understanding the organizational impact [31-33]. Kooij et al [31] focused on understanding how organizational factors impact the implementation process of patient portals by focusing on several hospital settings and identified a number of facilitators and barriers. Their findings are similar to what we found in terms of a lack of perceived value and willingness to change by health care providers. These findings are also similar to those of Koivunen et al [32] and McGinn et al [33]. However, these latter studies focus on understanding the facilitators and barriers from the perspectives of different stakeholders (providers, managers, IT providers, and patients), whereas our focus has been on understanding the internal organizational facilitators and barriers that impact the planning and implementation stages of a patient portal system. Our intent was to understand the internal drivers of change and how decisions were made to support implementation.

In this study, we found that incorporating project management principles led to a more focused scope that was aligned with the limited funding. These principles (vision and mission, objectives, standards of engagement, intervention and execution, organizational alignment, and measurement and accountability) allowed the implementation team to maintain a detailed record and track progress in real time. The Working Group incorporated project management principles for each project stage (planning, development, implementation, optimization, evaluation, and adoption). Studies by Richer et al [34] and Aubry et al [35] confirm that projects that have the potential to impact organizational change provide benefit not only by incorporating project management principles but also by establishing a project management office and central decision making to improve resource allocation.

According to the US Office of the National Coordinator for Health Information Technology, *the challenge of narrowing a large field of available options to a manageable number of vendors can be daunting, but it is a critical step* [36]. It is not only about selecting the best vendor but also selecting a vendor that *is willing to make commitments to gain new business* [37]. In this study, we were able to gain an in-depth understanding of how the vendor selection process evolved. The vendor section was based on the perceived ability of Epic to help in the future as a partner to deploy the provincial clinical information system.

### Implications and Lessons Learned

**Figure 8:**
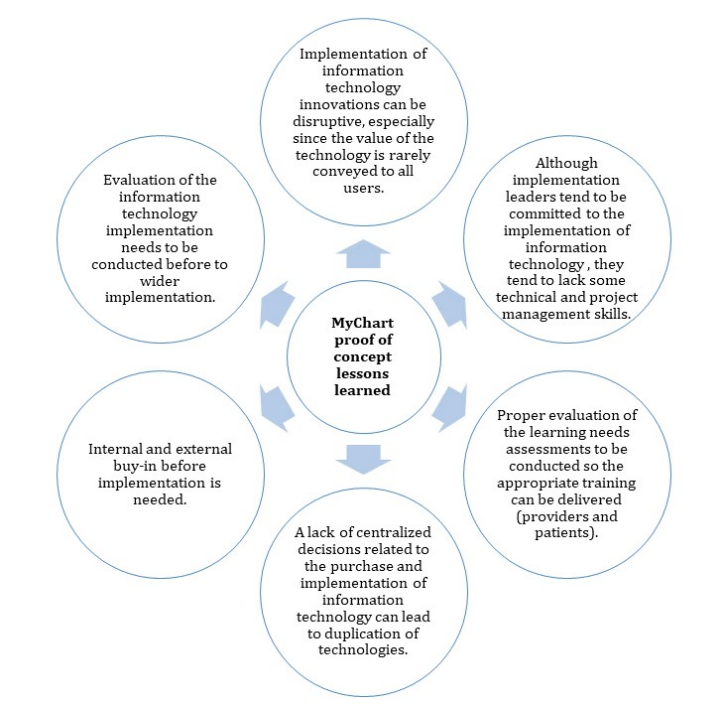
MyChart proof of concept lessons learned. IT: information technology.

Although many aspects of what occurred during the MyChart PoC processes are considered common with the implementation of health ITs, there are several features with broad implications for planning and delivering patient portals in a large public health care system that were evident during the MyChart PoC in Alberta, as described in Figure 8.

First, the implementation of IT innovations can be disruptive, especially as the value of the technology is rarely conveyed to all users [38,39]. *Value, by definition, includes both costs and benefits* [40]. This is confirmed by several reviews [40-43]. One review concluded, the *human element* is critical to health IT implementation [42]. Although these IT innovations are seen as a *key component of health care transformation to reduce costs and improve quality*, their adoption *proceeds at a snail’s pace* [40]. Furthermore, there tends to be a lack of properly *selecting which IT projects are of most value* [43]. Selecting the right technology requires an understanding of the value that the technology has the capacity to deliver and the ability to convey that value to all end users [44,45]. As the literature points out, health IT implementation should be considered as a complex intervention and that *complex interventions may work best if tailored to local circumstances rather than being completely standardized* [46]. Second, although implementation leaders tend to be committed to the implementation of IT, they tend to lack some technical and project management skills [47]. These types of implementation require appropriate skills to ensure successful utilization [46,48]. Third, proper evaluation of the learning needs assessments to be conducted so that appropriate training can be delivered (providers and patients) [49-52]. Quick presentations or Web-based technical training did not seem to resonate with the health care providers during the MyChart PoC. Fourth, a lack of centralized decisions related to the purchase and implementation of IT can lead to duplication of technologies [47,53-55]. Fifth, internal and external buy-in before implementation is needed [47,56,57]. Studies have shown that without the proper buy-in from providers, patients lack information about the opportunities to access their medical records [58]. Without the proper buy-in, providers and decision makers tend to doubt the usefulness of the technology [39]. Sixth, evaluation of IT implementation needs to be conducted before wider implementation [59,60]. Furthermore, evidence from pilots is rarely used when planning the implementation on a wide scale [60]. According to Houston et al [61], *the domain of Health Informatics is at risk for too rapid implementation as external pressures continue to promote adoption*. Although decisions are made to invest in the purchase and implementation of patient portals, the evidence of utilization and adoption has produced mixed results [41,62,63]. There is a rapid desire to implement patient portals without clear evidence that these technologies have the desired impact on the target populations in terms of effectiveness and safety [61].

The findings in this study highlight the importance of understanding internal organizational and decision-making approaches that have the capacity to hinder the planning and implementation of patient portals. It shows how organizations decide on IT investments, the intricacies of the decision processes, and factors affecting decisions at each stage to provide better future preparations for the successful implementation of technologies. Furthermore, our findings also document the effect of various social and political spheres on the development and implementation of MyChart and identify key factors that government and health care organizations may wish to consider before funding IT in health care.

### Limitations

There are several limitations to this study. First, we were unable to receive archival information from AH to describe their patient portal development process in its entirety. Second, although many efforts were made, we were unable to interview any AHS individuals who were considered IT technical experts.

### Conclusions

Implementing patient portals is a complex undertaking, as “it’s much more about the people who are using it that actually can make an impact on care” (AHS senior-level participant 6). The results of this study document the effect of various social and political spheres of influence on the development and implementation of MyChart and identify key factors that government and health care organizations may wish to consider before funding IT in health care. This study supports decision makers in understanding and managing the necessary organizational change and managing the individual expectations when implementation technologies have different types of usage by different groups.

## Abbreviations

AH: Alberta Health
AHS: Alberta Health Services
ANP: Alberta Netcare Portal
EHR: electronic health record
EMR: electronic medical record
IT: information technology
PCNs: primary care networks
PHR: personal health record
PoC: proof of concept
RFPs: request for proposals
